# Severe long-delayed malaria caused by *Plasmodium malariae* in an elderly French patient

**DOI:** 10.1186/s12936-021-03870-4

**Published:** 2021-08-05

**Authors:** Anthony Marteau, Elise Ouedraogo, Guillaume Van der Meersch, Mohammad Akhoundi, Berenice Souhail, Yves Cohen, Olivier Bouchaud, Arezki Izri

**Affiliations:** 1 Parasitology-Mycology Department, Avicenne Hospital, AP-HP, 125, route de Stalingrad, 93009 Bobigny cedex, France; 2 Infectious diseases Department, Avicenne Hospital, AP-HP, Bobigny, France; 3 Service de Réanimation Médico-Chirurgicale, Hôpital Avicenne, Assistance Publique-Hôpitaux de Paris, Bobigny, France; 4 Unité des Virus Émergents (UVE: Aix-Marseille Univ-IRD 190-Inserm 1207-IHU Méditerranée Infection), Marseille, France

**Keywords:** Imported malaria, Severe malaria, *Plasmodium malariae*.

## Abstract

**Background:**

*Plasmodium malariae* is the cause of the rare but severe form of malaria that sometimes affects individuals travelling to malaria-endemic regions. This report presents the unique case of a patient exhibiting severe malaria symptoms caused by *P. malariae* with no record of recent travel to any malaria-endemic areas.

**Case presentation:**

An 81-year-old French woman was admitted to the emergency department with sustained fever and severe weakness for the past 5 days. She suffered from anaemia, thrombocytopenia, confusion, somnolence, pulmonary complications, and hypoxaemia. In the absence of any concrete aetiology that could explain the fever together with thrombocytopenia, physicians suspected malaria as a probable diagnosis.

The LAMP-PCR and lateral flow test confirmed the presence of malaria parasite, *Plasmodium* sp. Microscopic examination (May-Grünwald Giemsa-stained thin blood smear) revealed the presence of trophozoites, schizonts, and gametocytes with 0.93 % parasitaemia. Conventional PCR amplification targeting 510 bp DNA fragment of small subunit ribosomal RNA (ssrRNA) and bidirectional sequencing identified the parasite as *Plasmodium malariae*. The travel history of this patient revealed her visits to several countries in Europe (Greece), North Africa (Tunisia and Morocco), and the West Indies (Dominican Republic). Of these, the latter was the only country known to be endemic for malaria at the time (three malaria parasite species were prevalent: *Plasmodium falciparum*, *Plasmodium vivax*, and *P. malariae*). The patient had most likely got infected when she visited the Dominican Republic in the summer of 2002. This time interval between the initial parasite infection (2002) till the onset of symptoms and its subsequent diagnosis (2020) is a reminder of the ability of *P. malariae* to persist in the human host for many years.

**Conclusions:**

This report highlights the persistent nature and ability of *P. malariae* to cause severe infection in the host even after a prolonged time interval.

## Background

Malaria is a serious public health problem caused by *Plasmodium* spp that is transmitted by the bite of female *Anopheles* mosquitoes [1]. With about 200 million annual cases and more than 400,000 deaths worldwide, it affects populations in more than 100 countries. Most countries in tropical and subtropical areas, especially in sub-Saharan Africa, South America, and Southeast Asia, are the worst affected [2–4]. Malaria is the most common cause of travel-associated tropical diseases among travellers [5]. Five species of malaria parasites (*Plasmodium falciparum*, *Plasmodium vivax*, *Plasmodium ovale*, *Plasmodium malariae* and *Plasmodium knowlesi)* can infect humans, of which *P. falciparum* and *P. vivax* are the most common. *Plasmodium falciparum* causes malaria with severe clinical symptoms [6]. Fever, chills, headache, fatigue, nausea, and vomiting are some of the most common clinical indications of the disease [7]. Most malaria-related deaths are caused by *P. falciparum*, but severe malaria cases have also been described with *P. vivax*, and to a lesser extent with *P. malariae*, *P. knowlesi* and *P. ovale* [8, 9].

*Plasmodium malariae* usually causes a mild form of malaria prevalent in some parts of Africa, South America, Asia, and the Western Pacific [10–13]. It has the longest incubation period among all *Plasmodium* species, ranging between 18 and 40 days, which sometimes even last for several years [14]. Infection is often asymptomatic, but in rare severe cases, the disease progresses rapidly and, in the absence of treatment, may lead to death within hours or days [9, 13].

This report describes the case of an elderly patient with no recent history of travel to a malaria-endemic country, but was diagnosed with a severe form of malaria caused by *P. malariae*.

## Case presentation

On June 3rd, 2020, an 81-year-old French woman was admitted to the emergency department of a Paris hospital, for continuous fever and severe weakness for 5 days. On arrival, she had a mild fever (38.5 °C). She had a past medical history of chronic obstructive pulmonary disease (COPD) due to smoking (60 packs/year) and depression. Her past travel itinerary revealed her trips to Greece (2000), Dominican Republic (2002), Morocco (2010), Greece (2013 and 2015), and more recently to Tunisia (2019).

Cell blood count examination revealed moderate anaemia (10.8 g/dL, N = 12–16 g/dL), thrombocytopenia (65*10^9^/L, N = 150–400*10^9^/L), lymphocytopenia (0.3*10^9^/L, N = 1–4*10^9^/L), and normal leukocyte count (5.5*10^9^/L, N = 4–10*10^9^/L). The biochemical analyses of blood showed a normal creatinine (83µmol/L, 45–84 µmol/L) and glucose level (6.5mmol/L), a pH of 7.45, and an imbalance in electrolytes (Na = 126mmol/L, CL = 93mmol/L, K = 4mmol/L, and plasma lactate = 3.3mmol/L). A liver function test revealed ALT (38U/L) within normal range but an elevated AST (92U/L) with subsequent high C-reactive protein levels (84 mg/L, N < 5 mg/L). The blood tests were repeated a few days later, which showed a sudden decrease of thrombocytopenia (35*10^9^/L, N = 150–400*10^9^/L) and an elevated level of C-reactive protein (165 mg/L, N < 5 mg/L). Urine analysis showed Na < 20 mmol/L, K = 50 mmol/L; Urea = 323 mmol/L; Creatinine = 8.8 mmol/L. The clinical examination showed difficulty in swallowing and crepitations in the right lung. There were no abnormal respiratory, urinary, or digestive indications. Three days later, the thoraco-abdomino-pelvic computed tomography (CT) scan revealed a centrilobular emphysema probably related to the earlier history of COPD. No pulmonary edema or hepatosplenomegaly was seen. As inhalation pneumopathy was suspected, presumptive treatment with antibiotics Ceftriaxone (2 g/day) and Metronidazole (1.5 g/day) was given for 7 days. The patient was transferred to the ICU Department of Avicenne Hospital (Bobigny, France) on June 9th 2020, due to deterioration in her health condition along with hypoxemia (89 %). The patient was given supplemental oxygen at 2 L/minute. The clinical examination revealed a fever (39 °C), hypotension (90/50mmHg), confusion, somnolence (Glascow Coma Scale = 14) without sensory-motor deficits, cold peripheries, and diarrhoea. The history of frequent travels, sustained fever, along with thrombocytopenia suggested signs of malaria disease.

**Fig. 1:**
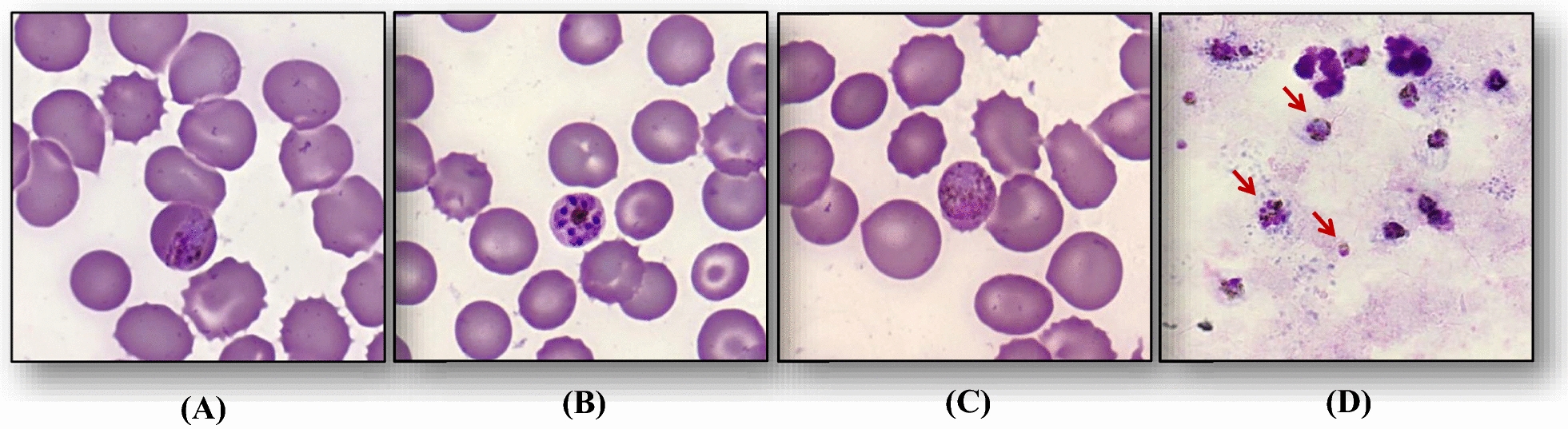
Giemsa-stained thin blood smears depicting Trophozoite (**A**), Schizont (**B**), and Gametocyte (**C**) of *Plasmodium malariae* together with thick blood smear (**D**) of mentioned forms (highlighted by red arrows) in our patient observed by microscopy under X1000 magnification

The LAMP-PCR (Alethia® Malaria, Meridian Bioscience) and lateral flow test (VIKIA® Malaria Ag Pf/Pan, Biomérieux, France) confirmed the presence of malaria parasite, *Plasmodium* sp. Microscopic examination (May-Grünwald Giemsa-stained thin blood smear) revealed the presence of trophozoites, schizonts, and gametocytes with 0.93 % parasitaemia. The particular band-form trophozoites, and other species-specific developmental stages of the parasite within RBC validated the presence of *P. malariae* (Fig. 1). Conventional PCR amplification targeting 510 bp DNA fragment of small 

subunit ribosomal RNA (ssrRNA) and bidirectional sequencing identified the parasite as *P. malariae* [15]. The patient was thus treated with chloroquine (10 mg/kg oral tablet in d1 and d2, and 5 mg/kg in d3) and 1 L/6 h IV perfusion of NaCl. The MGG-stained thick blood smears were examined on day 3 and day 7, after being shifted from ICU to a general ward, and a favourable evolution was observed within 5 days, with negative microscopy on d7.

## Discussion and conclusion

France has one of the highest number of malaria cases reported in returned travellers, with about 5000 cases per year [4, 16]. Around 95 % of the malaria cases are observed in people returning from malaria-endemic sub-Saharan African countries [4]. *Plasmodium falciparum* alone is responsible for 85 % of malaria cases and makes up about 12–14 % of severe cases of malaria [4, 17]. Nevertheless, some reports of local cases have also been documented in metropolitan France [18–20].

*Plasmodium malariae* is a less frequent but widely distributed species worldwide. Although it does not cause a relapse, it can latently persist in the human host for many years. The parasite can survive in the human host at low-level parasitaemia for decades that sometimes manifest in chronic morbidity [21, 22]. This low parasitaemia is likely due to the fewer merozoites released from schizonts, extended duration of the erythrocytic stage of the parasitic life cycle, preferential invasion of erythrocytes, and host immunity [23]. Severe complications in malaria are rare in patients infected with *P. malariae* (known only in about 2 % cases) [9]. Severe anaemia, cerebral malaria, convulsions, pulmonary issues, acute kidney injury, and renal impairment are the common complications found in patients infected with *P. malariae* [23–25]. The renal impairment can happen due to haemodynamic dysfunction or overt immune response and can be manifested as proteinuria, microalbuminuria, or urinary casts [26]. Malaria caused by *P. malariae* is usually seen in older people [22]. The case study presented here is of an elderly woman who suffered from fever, confusion, somnolence, pulmonary issues, and hypoxaemia. All life cycle stages of *P. malariae* were visible in blood smear, indicating that no splenic sequestration occurred in the patient (Fig. 1). According to World Health Organization guideline 2001 and French recommendation 2017, the patient’s symptoms of somnolence and confusion were considered as a case of severe malaria. A thorough literature search revealed 7 severe cases of malaria caused by *P. malariae* reported from Bangladesh, Indonesia, Ivory Coast, Italy, Nigeria, Senegal, and the USA [22, 24, 25, 27–30]. Out of these, four cases were of returned travellers from malaria-endemic regions, and the rest being residents of malaria-endemic areas [27, 28]. It is thus highly likely that this patient was probably infected in Punta Cana (Dominican Republic) where she stayed for 15 days. Despite the re-introduction of *P. vivax* in southern Europe [31], it is unlikely that she was infected in Greece. Furthermore, Morocco and Tunisia have been malaria-free countries since 2010 and 2015, respectively [32, 33]. At least three species, *P. falciparum*, *P. vivax*, and *P. malariae* have been reported from the Dominican Republic [34–36]. In addition, the long time interval between the initial malaria infection in the Dominican Republic (as the sole known endemic region of malaria among travelled countries) in 2002 and diagnosis of the disease in 2020 is a reminder of the competency of *P. malariae* to persist in the human host for many years. The patient inhabited in a city close to the Charles de Gaulle Airport (one of the overcrowded airports in the world with the highest number of passenger transfers) in Paris which suggested the possibility of airport malaria [37]. Nevertheless considering a significant distance between the residence of the patient and Charles de Gaulle Airport (about 12 km distance), this option was only considered secondary. She never took any malaria prophylactic measures. This report highlights the importance of collecting detailed travel itineraries from travellers or immigrants coming from malaria-endemic regions and emphasizes the requirement of awareness among clinicians for timely diagnosis and effective treatment.

In patients with severe malaria, early diagnosis is crucial in preventing complications that can be life-threatening. In addition, to improve survival rates, treatment with anti-malarial drugs and supportive measures should be initiated as early as possible. In the present case, cumulative clinical, biochemical, parasitological, and molecular test results allowed the physicians to diagnose the disease with minimal time delay. Subsequently, the patient was treated with chloroquine, the WHO-recommended medication for malaria due to *P. malariae* [38, 39]. Clinicians should be aware of the potential complications associated with *P. malariae* infection and that the early diagnosis is the key to save lives.

## Data Availability

Not applicable.
